# Supplementary figures and images for: Altered Calcium Influx Pathways in Cancer-Associated Fibroblasts
Source: Biomedicines. 2021 Jun 16;9(6):680. doi: 10.3390/biomedicines9060680 (PMC8234491; doi:10.3390/biomedicines9060680)

Additional File 2: Fig. S1. Sadras et al.

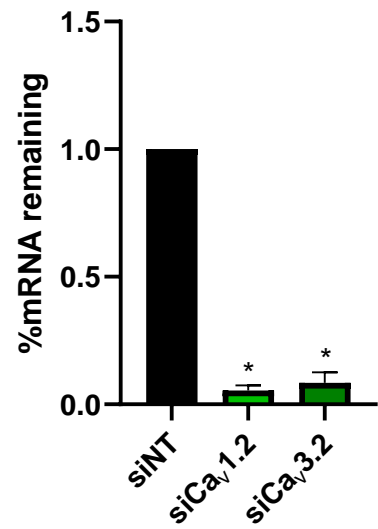

Supplement: Supplementary file 1 [file biomedicines-09-00680-s001.zip › Supplementary figures/Additional File 2. Figure S2.pdf]
